# Supplementary material for: A New Biocontrol Agent Bacillus velezensis SF334 against Rubber Tree Fungal Leaf Anthracnose and Its Genome Analysis of Versatile Plant Probiotic Traits
Source: J Fungi (Basel). 2024 Feb 17;10(2):158. doi: 10.3390/jof10020158 (PMC10890420; doi:10.3390/jof10020158)
Supplement: Supplementary file 1 [file jof-10-00158-s001.zip › jof-2863720-supplementary.pdf]

A New Biocontrol Agent *Bacillus velezensis* SF334 Against Rubber Tree Fungal Leaf Anthracnose and Its Genome Analysis of Versatile Plant Probiotic Traits

Muyuan Wang [1,†], Yikun Zhang [1,†], Haibin Cai[2], Xinyang Zhao[3,], Zhongfeng Zhu[1], Yichao Yan[1], Ke Yin[1], Guanyun Cheng[1], Yinsheng Li[1], Gongyou Chen[1,4], Lifang Zou[1, 4, *], Min Tu[2, 5, *]

Table S1. The plant growth promotion-associated genes in *B. velezensis* SF334

Gene ID	KO	Gene	Function	Class
SF334GL002344	K01695	*trpA*	tryptophan synthase alpha chain	Phenylalanine, tyrosine and tryptophan biosynthesis
SF334GL003147	K14155	*patB, malY*	cysteine-S-conjugate beta-lyase	Biosynthesis of secondary metabolites
SF334GL002056	K00128	*aldh*	aldehyde dehydrogenase (NAD+)	Tryptophan metabolism
SF334GL002972	K00128	*aldh*	aldehyde dehydrogenase (NAD+)	Tryptophan metabolism
SF334GL004032	K00128	*aldh*	aldehyde dehydrogenase (NAD+)	Tryptophan metabolism
SF334GL002345	K01696	*trpB*	tryptophan synthase beta chain	Phenylalanine, tyrosine and tryptophan biosynthesis
SF334GL002347	K01609	*trpC*	indole-3-glycerol phosphate synthase	Phenylalanine, tyrosine and tryptophan biosynthesis

Table S2. General features of genomes of *B. velezensis* SF334, *B. velezensis* FZB42, *B. velezensis* SQR9, *B. amyloliquefaciens* DSM7, and *B. subtilis 168*

General features	*B. velezensis* SF334	*B. velezensis* FZB42	*B. velezensis* SQR9	*B. amyloliquefaciens* DSM7	*B. subtilis* 168
Genome size (bp)	4,078,641	3,918,596	4,117,023	3,980,199	4,227,167
GC content	46.5%	46.48%	46.10%	46.08%	43.49%
Coding density (%)	89.33	89.026	89.68	88.54	88.33
Protein coding sequences (CDS)	4,142	3,888	4,111	4,220	4,388
tRNA	86	89	72	94	86
5s rRNA	9	10	7	10	10
16s rRNA	9	9	7	10	10
23s rRNA	9	10	7	10	10
Repeat region	30	31	20	43	38

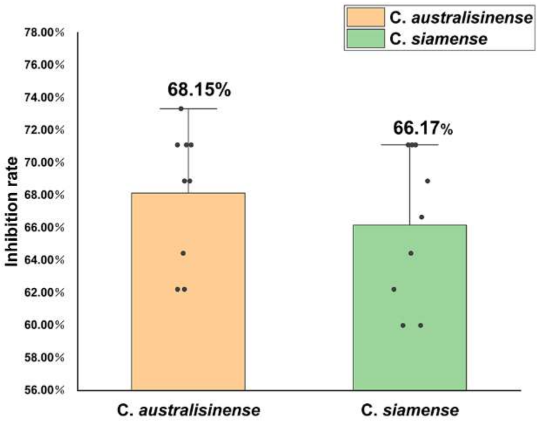

Figure. S1. Antagonistic effect of strain SF334 against *C. siamense* and *C. australisinense,* which are major pathogens causing leaf anthracnose of rubber trees in the Hainan province of China. The average inhibition rates of SF334 against *C. siamense* and *C. australisinense* were calculated.

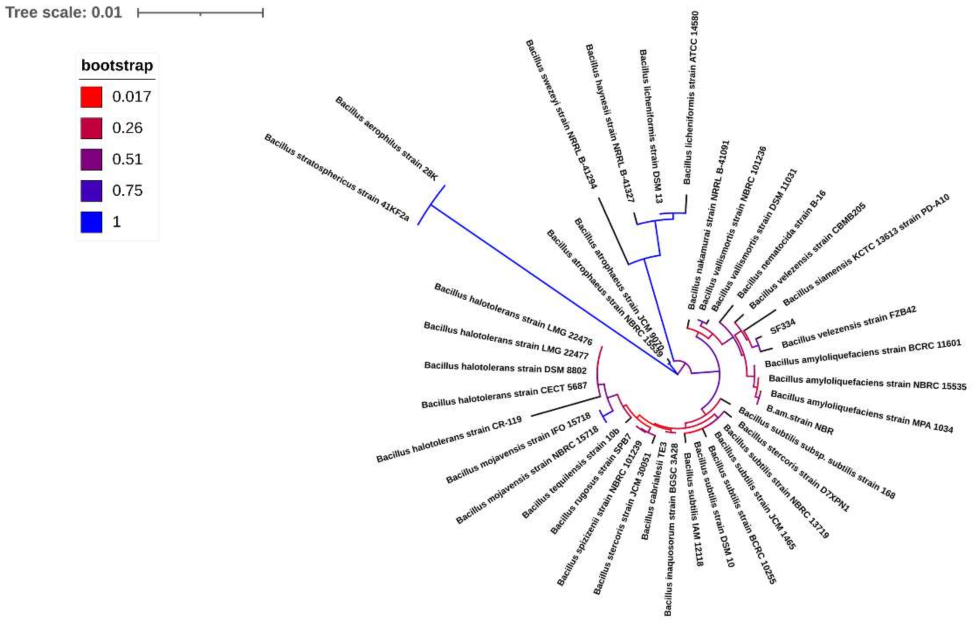

Figure S2. The phylogenetic tree of strain SF334 based on *16S rDNA* sequences constructed through the TYGS platform. The sequence of *16s rRNA* of 39 strains with the lowest E-value in the National Center for Biotechnology Information (NCBI) database were selected as the reference sequences. The results were finally imported into MEGA 11 soft to construct a phylogenetic tree by the NJ (1200 bootstrap) method.

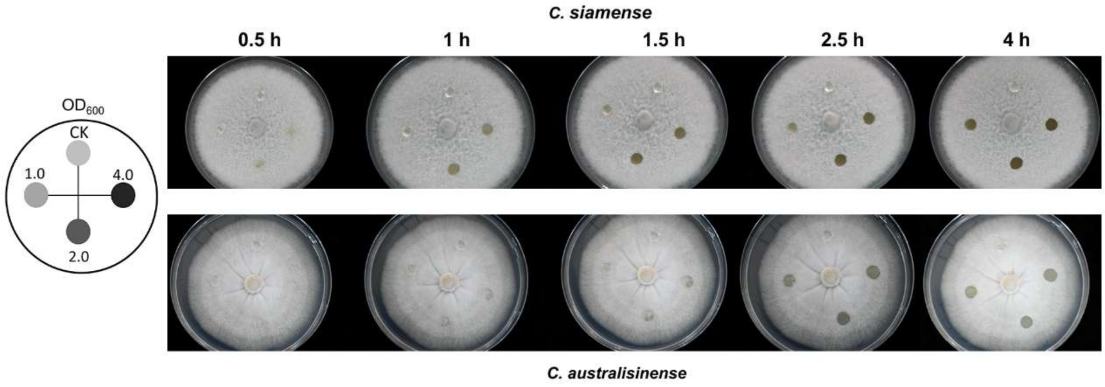

Figure S3. Observation of the hyphal lysis of *C. siamense* and *C. australisinense* when interacting with the CSs of *B. velezensis* SF334 on PDA plates. The mycelium of *C. siamense* and *C. australisinense* were inoculated with the indicated bacterial concentration for 0.5 h, 1h, 1.5 h, 2.5 h and 4 h.

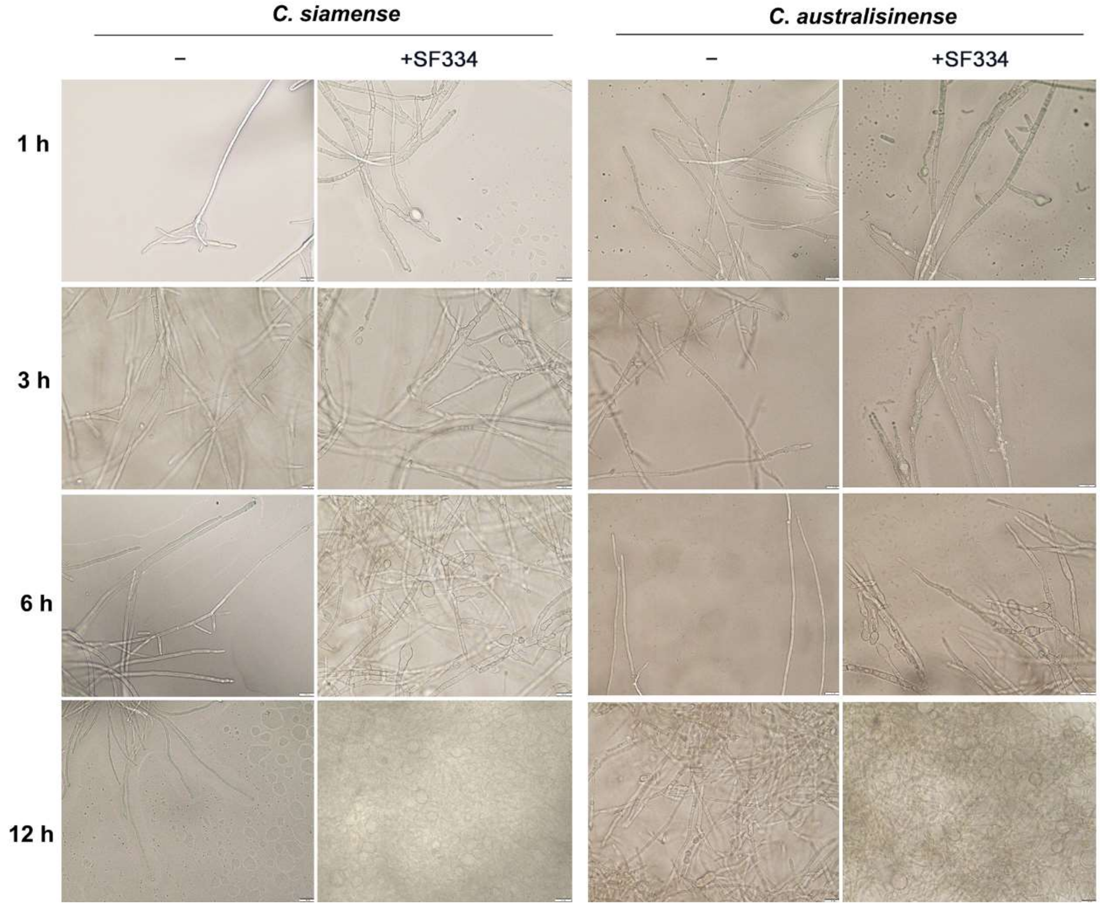

Figure S4. Observation of mycelium morphology of *C. siamense* and *C. australisinense* when interacting with the CSs of *B. velezensis* SF334 for 1 h, 3 h, 6 h, and 12 h in PDB medium using an optical microscope. Scale bar, 10 μm.

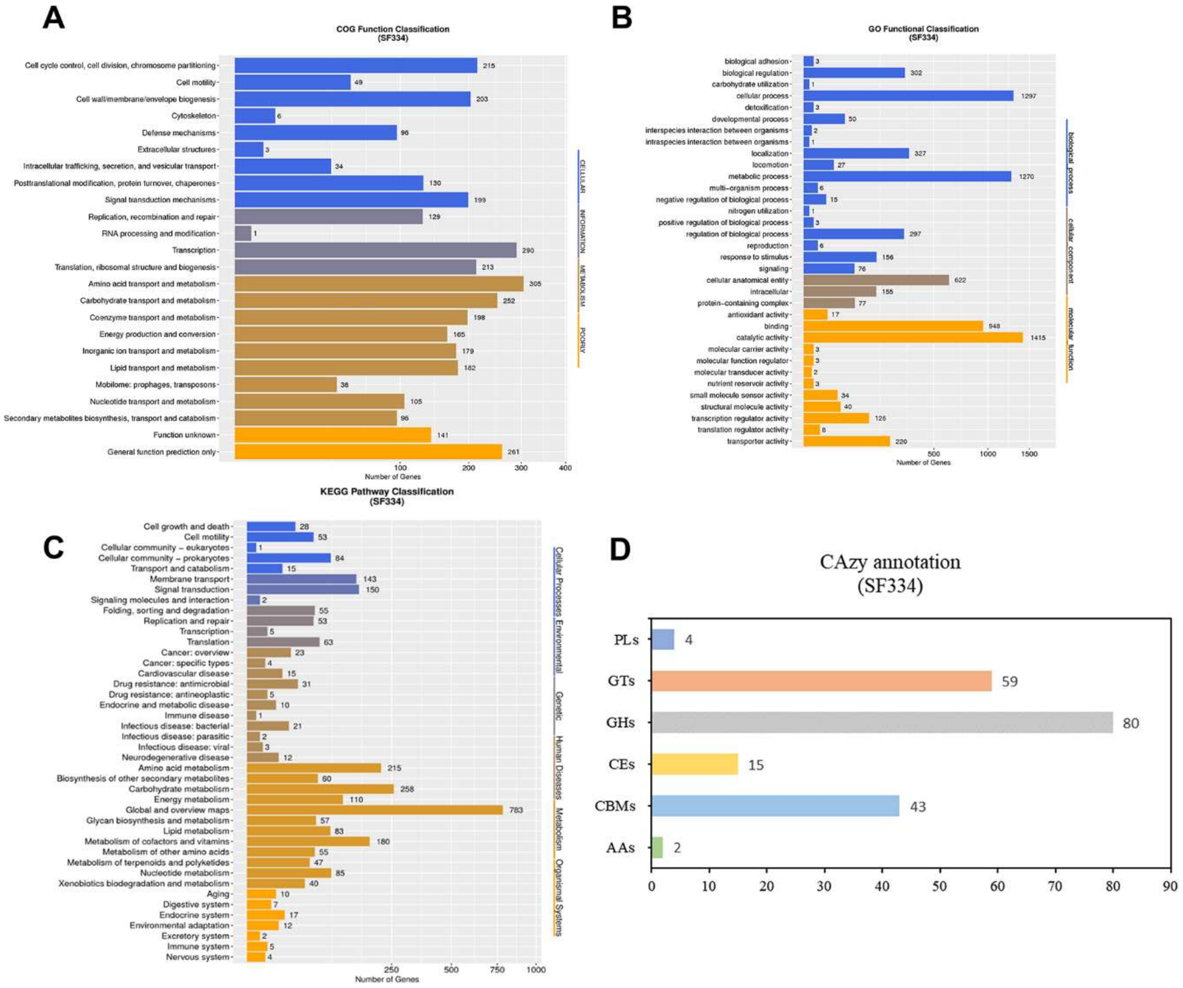

Figure S5. Genomic analysis of *B. velezensis* SF334. The COG analysis (A), GO analysis (B), KEGG pathway analysis (C) and CAZy annotation (D) of *B. velezensis* SF334

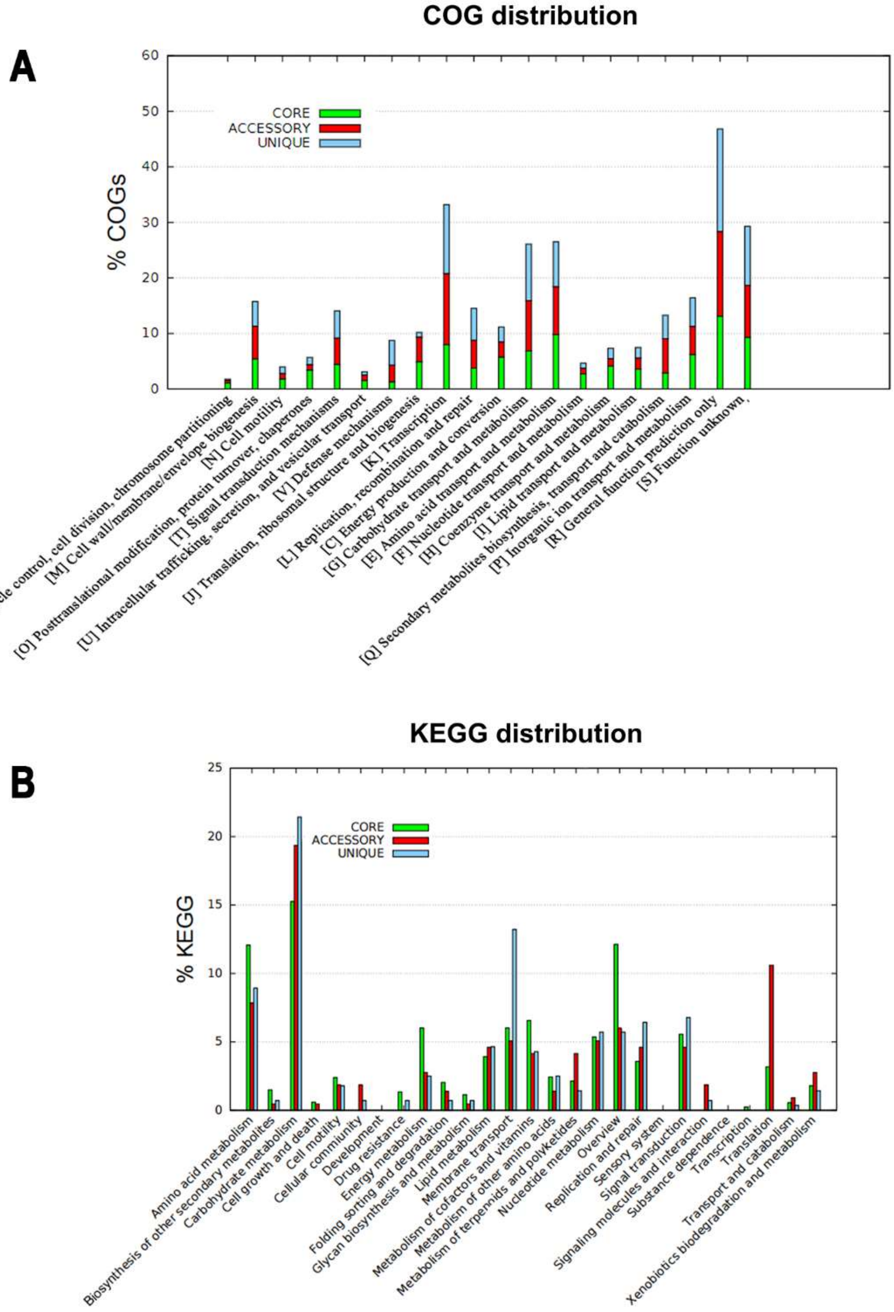

Figure S6. Comparative genomic analysis of *B. velezensis* SF334 with four other related *Bacillus* species. The COG analysis (A) and KEGG pathway analysis (B) of *B. velezensis* SF334 with *B. velezensis* FZB42, *B. velezensis* SQR9, *B. amyloliquefaciens* DSM7, and *B. subtilis* 168. Green, red and blue boxes indicate core genes, accessory and unique genes between five strains.
